# Experimental data on the characterization of hydroxyapatite synthesized from biowastes

**DOI:** 10.1016/j.dib.2019.104485

**Published:** 2019-09-11

**Authors:** J.K. Abifarin, D.O. Obada, E.T. Dauda, D. Dodoo-Arhin

**Affiliations:** a Department of Mechanical Engineering, Ahmadu Bello University, Zaria, Nigeria; b Department of Metallurgical and Materials Engineering, Ahmadu Bello University, Zaria, Nigeria; c Department of Materials Science and Engineering, University of Ghana, Legon, Ghana; d Institute of Applied Science and Technology, University of Ghana, Legon, Ghana

**Keywords:** Animal bones, Calcination, Microstructure, Infrared region

## Abstract

The purpose of the dataset is to present the morphological features, elemental composition and functional groups of hydroxyapatite (HAp) synthesized from non-separated biowastes (animal bones) by a modified facile heat treatment method up to a maximum temperature of 1100 °C. The synthesized powders were characterized using scanning electron microscopy (SEM) equipped with electron dispersive X-ray analysis (EDX) and Fourier transform infrared spectroscopy (FTIR). These evaluations were to reveal the surface features, elemental composition and identify the functional groups of the synthesized powders. After heat treatment of the raw biowastes to 900 °C, 1000 °C, and 1100 °C (regime of heat treatment), the morphological features of the samples exhibited a more densely packed microstructure at the highest sintering temperature (1100 °C). The elemental composition as evaluated by EDX on a weight and atomic basis for all samples provided information on the calcium to phosphate transforms into apatite with a Ca/P ratio of 3.60, 2.04, 2.50 and 2.32 wt % and 2.79, 1.58, 1.94 and 1.78 at. % respectively for raw biowastes (RB) to sintered samples (HA-900, HA-1000 and, HA-1100 °C). The FTIR data showed phosphate and hydroxyl peaks in the thermally treated samples and all the samples produced characteristic stretching modes of O–H bands at about 3417 cm^−1^ which are noticed in all FTIR spectra of HAp.

**Table:** Specifications Table

Subject	Engineering
Specific subject area	Biomedical Materials: synthesis and characterization
Type of data	Table Image Chart Graph Figure
How data were acquired	SEM/EDX and FTIR techniques
Data format	Raw Analysed Images Tables Plots
Parameters for data collection	Hydroxyapatite was produced from the calcination of clean animal bones at 900 °C, 1000 °C, 1100 °C for a dwell time of 2 h.
Experimental features	1. The animal bones were thoroughly cleaned to remove the protein externally by soaking for 24 h, washing with pure water and boiling for 3 h 2. The cleaned bones were rinsed in warm water, and dried at 150 °C in an electric oven for 8 h. 3. Calcination was conducted under atmospheric condition using an electric furnace at 900 °C at a ramp rate of 5 °C/min with 2 h of soaking time 4. The calcined powders were crushed with a metallic mortar and pestle and sieved through a 300 μm mesh sieve to obtain a fine powder prior to characterization.
Data source location	Department of Mechanical Engineering, Ahmadu Bello University, Zaria, Nigeria
Data accessibility	With the article

**Table:** 

**Value of the data** • The dataset can be used to monitor changes in the properties of hydroxyapatite derived biomaterials at the microscale • Researchers working on biomaterials and biowaste valorization can benefit hugely from the data • The FTIR data provided in this article provides an indirect evaluation of the synthesized hydroxyapatite, showing the most characteristic chemical groups in the FTIR spectrum of synthesized HAp such as PO_4_^3−^, OH-, CO_3_^2−^. • Data obtained in this study would be useful for future investigation on the industrial applicability of biowastes derived hydroxyapatite

## 1 Data

**Fig. 1:**
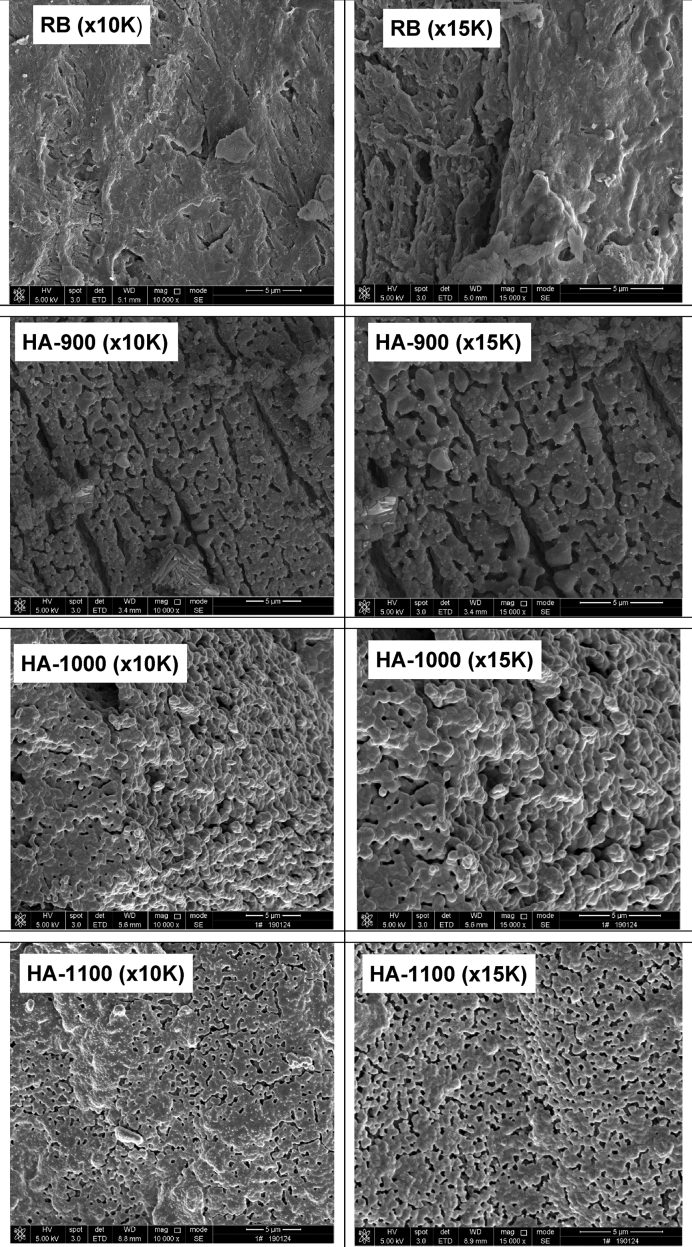
SEM micrographs of RB, HA-900, HA-1000 and HA-1100 at different magnifications.

**Fig. 2:**
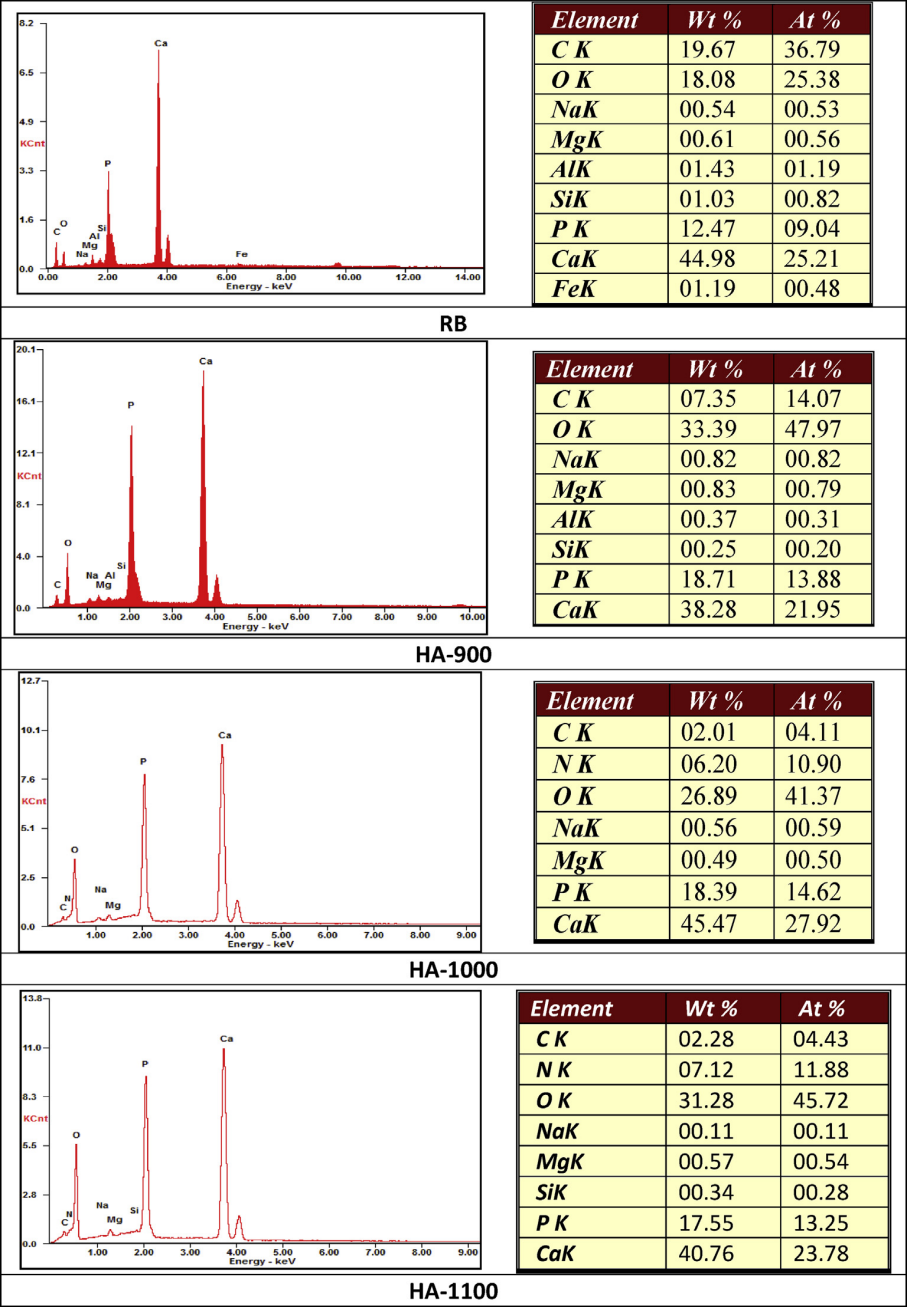
EDS spectra of RB, HA-900, HA-1000 and HA-1100.

The dataset presented in this article for SEM and FTIR analyses as shown in Fig. 1, Fig. 2 are for synthesized hydroxyapatite subjected to various sintering temperatures (900 °C, 1000 °C, 1100 °C). The morphology of samples in Fig. 2 is at a magnification of 10.0 and 15.0 kx. The elemental composition and FTIR summary tables (Table 1a, Table 1ba and 1b and Table 2) show Ca/P ratios and obtained functional groups which provides useful information about the composition of samples and the location of peaks, their intensities, width and shape in the required wave number range. Raw data for FTIR spectrogram is presented as supplementary files.

**Table 1a:** Calcium/Phosphate ratio (Wt %).

Sample Code	RB	HA-900	HA-1000	HA-1100
Calcium (Wt %)	44.98	38.28	45.11	40.76
Phosphorus (Wt %)	12.47	18.71	18.00	17.55
Ca/P (Wt %)	3.60	2.04	2.50	2.32

**Table 1b:** Calcium/Phosphate ratio (At%).

Sample Code	RB	HA-900	HA-1000	HA-1100
Calcium (At%)	25.21	21.95	27.57	23.78
Phosphorus (At%)	9.04	13.88	14.23	13.25
Ca/P (At %)	2.79	1.58	1.94	1.79

**Table 2:** Wavenumbers, chemical groups and description of the FT-IR spectrum of RB, HA-900, HA-1000 and HA-1100.

Sample code and Wavenumber (cm^−1^)				Chemical group	Description	References
RB	HA-900	HA-1000	HA-1100			
563, 601	570, 601	570, 601	570, 601	PO_4_^3−^	bending mode	[3], [4], [5], [12], [13], [14]
671, 3417	632, 3417	632, 3417	632, 3417	OH^−^	OH^−^ ions prove presence of HA	[3], [4], [7], [12], [13], [14]
1033, 1134	1049, 1095	1049, 1095	1049, 1095	PO_4_^3−^	Antisymmetric stretching mode	[1], [3], [4], [5], [9], [12], [13], [14]
1412, 1466, 1643,	1411, 1465, 1550, 1643, 2152, 2345	1411, 1465, 1643, 2152, 2345	1411, 1465, 1643, 2553	CO_3_^2−^	Substitutes phosphate ion, B-type HA (i.e. CO_3_ substituting for PO_4_) is formed	[2], [6], [7], [8], [9], [12], [13], [14]
2862, 2924	2931	2924	2924	Adsorbed water	Under influence of thermal treatment, absorption band becomes narrower	[2], [8], [9], [12], [13], [14]

The surface morphology of the samples after sintering at 900 °C, 1000 °C and 1100 °C at different magnifications (10.0 and 15.0 kx) is shown in Fig. 1 and the corresponding EDS spectra is shown in Fig. 2. Close observation of the SEM images reveal increasingly dense microstructure as a function of increased sintering temperature. The densification of hydroxyapatite particles as revealed by SEM increases as sintering temperature increase. Samples sintered at 900 °C reveal fine grains which are not closely packed. Samples sintered at 1000 °C show some grain growth tendencies which can be attributed to re-crystallization phase with porosities embedded between the large grains. The sample sintered at 1100 °C show grain growth, closely packed particles as well as decreasing pore sizes and density. In addition, the micrographs in Fig. 1 show morphologies for all samples with fine particles and reduced pore size details (magnification 10.0 kx). With an increase to 15.0 kx (b), it is observed that the particles are clustered with more pore size details reavealed. From the EDX spectra as depicted in Fig. 2, the characteristic peaks of Ca, P and O are present with the atomic and weight percentages which provides the mean relative calcium to phosphate ratios as shown in Table 1a, Table 1ba and 1b.

Table 1a, Table 1ba and 1b represents the elemental composition of the raw biowastes and synthesized hydroxyapatite with their corresponding calcium to phosphate (Ca/P) ratios obtained by energy dispersive X-ray analysis (EDX). Weight percentage (wt%) and atomic percentage (at%) were considered to give Ca/P approximations. From the data (Table 1a, Table 1ba and 1b), calculated Ca/P ratios for sample HA-900 were 2.04 and 1.58 for weight and atomic percentages respectively. Comparatively, the atomic Ca/P ratio (1.58) of HA-900 was the closest to stoichiometric Ca/P ratio of hydroxyapatite (1.67) amongst all the hydroxyapatite samples investigated in this study.

**Fig. 3:**
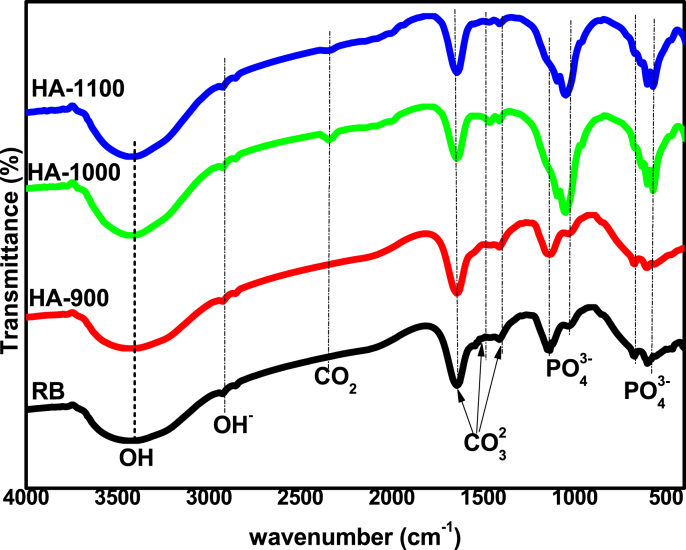
FT-IR spectrogram of RB, HA-900, HA-1000 and HA-1100.

Fig. 3 shows the Fourier Transform Infrared (FT-IR) spectrogram of synthesized HAp before and after sintering at different temperatures with further description in Table 2. Typical characteristic bands corresponding to the carbonate groups of the CaCO_3_ component of hydroxyapatite are located around 1411 cm^−1^ and 1465 cm^−1^. The sintered samples showed small frequency bands around 2345 cm^−1^ and 2353 cm^−1^ ascribed to the release of CO_2_ during heat treatment [10]. The initial formative indicator of hydroxyapatite at all point of sintering (900 °C, 1000 °C, 1100 °C) was in the form of a pronounced broad band around 1000 cm^−1^ –1100 cm^−1^ which can be ascribed to asymmetric stretching mode of vibration of PO_4_ group. However, as noticed for the raw biowastes bone, a small band noticed at 1033 cm^−1^ indicates a deficient asymmetric stretching mode of vibration of PO_4_ group that was made pronounced by heat treatment. Also, the band between 570 cm^−1^ – 565 cm^−1^ for all the samples corresponds to symmetric P–O stretching vibration of PO_4_ group [11]. The prominent bands at 3417 cm^−1^ for all samples is due to the vibratory stretching of OH group of HAp while the bands at 1643 cm^−1^ for all samples were attributed to adsorbed water molecules [1]. All the samples produced typical stretching modes of O–H bands at about 3417 cm^−1^ characteristic of FT-IR spectrogram of HAp. From the FT-IR spectrogram, differences between the spectrum of the raw and heat treated hydroxyapatite samples can be noticed for the bands at 1411 and 1465 cm^−1^ for HA-1000 and HA-1100 samples as compared to the bands ascribed to RB and HA-900 samples. These differences are indicative of the degree of carbonate ion substitution. The bands at 1643 and 3417 cm^−1^ for all samples with broader bands for RB and HA-900 samples correspond to the disappearance of absorbed water after sintering [15], [16].

## 2 Experimental design, materials, and methods

**Fig. 4:**
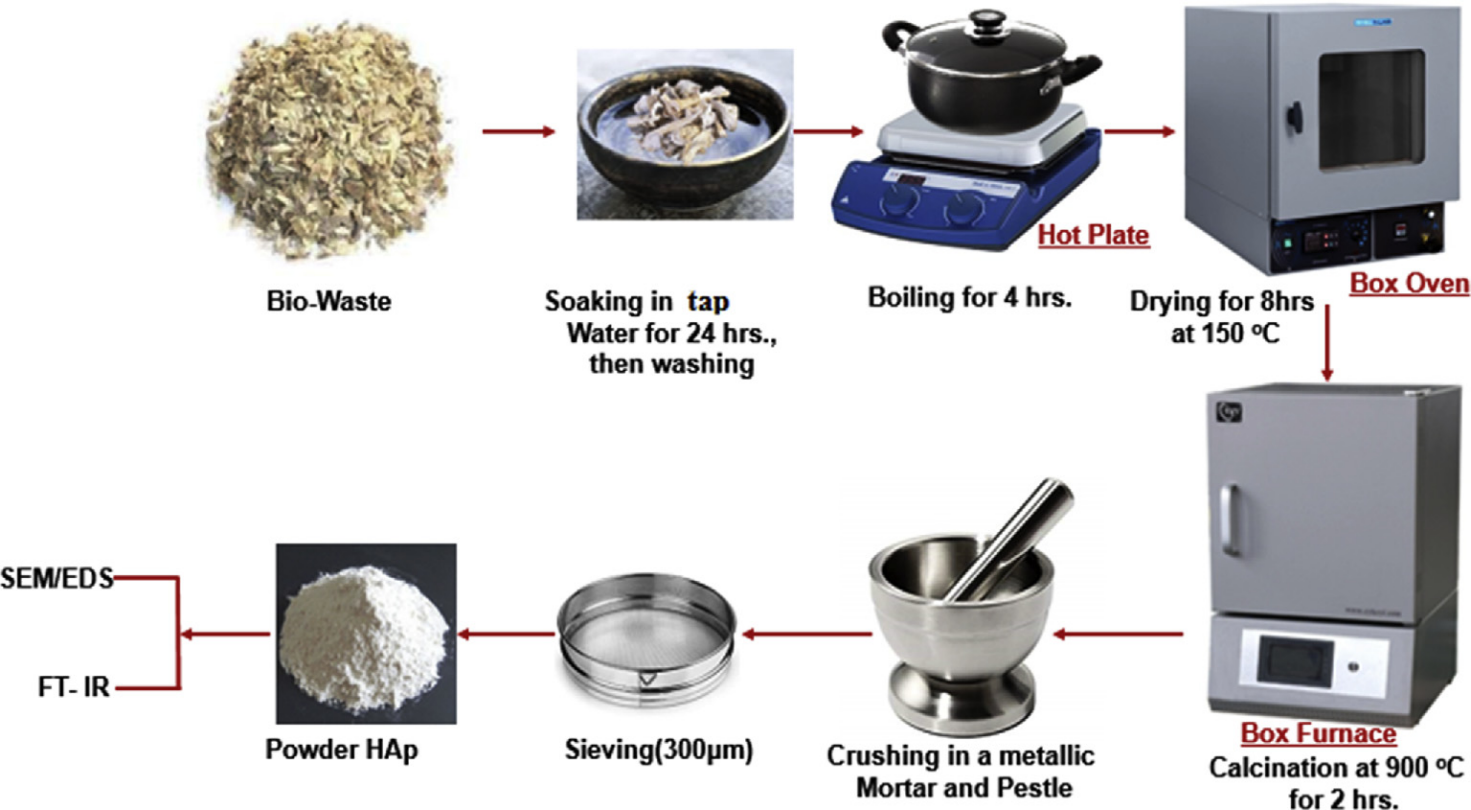
Schematic of hydroxyapatite preparation and characterization techniques.

The biowastes (animal bones) were obtained from an Abattoir in Zaria, Nigeria. The as-received bones were cleaned thoroughly to remove the protein externally by soaking for 24 h, washing with tap water and boiling for 3 h. This was followed by rinsing in warm water before drying at 150 °C in an electric oven for 8 h. The as-produced powders were calcined under atmospheric condition using an electric furnace at 900 °C at a ramp rate of 5 °C/min with 2 h of soaking time. The calcined powders were crushed with a metallic mortar and pestle and sieved through a 300 μm mesh sieve to obtain a fine powder prior to characterization. Fig. 4 shows a schematic of the experimental procedure for the synthesized hydroxyapatite. The produced hydroxyapatite powders were further sintered at 1000 °C and 1100 °C to investigate the effects of sintering temperature on the morphological features and retention of functional groups relating to produced hydroxyapatite. The nomenclature of samples synthesized and analysed are: RB (raw biowaste), HA-900 (samples sintered at 900 °C), HA-1000 (samples sintered at 1000 °C) and HA-1100 (samples sintered at 1100 °C).

The morphology of the samples was carried out on an ultra-high vacuum and high resolution MAIA3 TESCAN scanning electron microscopic—energy dispersive X-ray analysis (SEM-EDX) operated at 5 kV. The samples were prepared by gold sputtering the surface of the samples using a low deposition rate. The functional groups present in the samples have been identified by FT-IR equipped with UATR sampling accessory in the range of 500–4000 cm^−1^. Samples were grinded and mixed with dried KBr using ceramic mortar and loaded into a sample holder mounted in the instrument.
